# Free immunoglobulin light chain (FLC) promotes murine colitis and colitis-associated colon carcinogenesis by activating the inflammasome

**DOI:** 10.1038/s41598-017-05468-w

**Published:** 2017-07-12

**Authors:** Junfan Ma, Dongyang Jiang, Xiaoting Gong, Wenwei Shao, Zhu Zhu, Weiyan Xu, Xiaoyan Qiu

**Affiliations:** 1 Department of Immunology, School of Basic Medical Sciences, Peking University Health Science Center, Beijing, 100191 China; 2 Key Laboratory of Medical Immunology, Ministry of Health, Beijing, 100191 China; 3 Department of Cardiology, Cardiovascular Disease Institute, Shanghai Tenth People’s Hospital, Tongji University School of Medicine, Shanghai, China

## Abstract

Numerous studies have demonstrated that free Ig light chain (FLC), a novel inflammation mediator, participates in many inflammatory diseases by activating mast cells and extending the survival of neutrophils. However, it remains unclear whether FLC is involved in colitis and colitis-associated colon carcinogenesis (CAC). In this study, we found a significant increase in FLC in murine models of DSS (Dextran Sulfate Sodium Salt)-induced colitis and CAC compared to controls. Peptide F991, a functional blocker of FLC, significantly attenuated colitis progression, which included abrogating the development of diarrhea and tumor burden, elevating survival rate, greatly reducing the infiltration of inflammatory cells (such as ROS^+^ active neutrophils), especially reducing tumorigenesis in CAC. Furthermore, we demonstrated that F991 inhibited the activation of the inflammasome by reducing the expression of cleaved caspase-1 and the maturation of IL-1β and IL-18. Altogether, our findings demonstrate that FLC can promote the pathogenesis of colitis and CAC and may be used as novel biomarker for the diagnosis of inflammatory bowel disease. Additionally, F991 may become a potential therapeutic option for colitis or colorectal cancer.

## Introduction

Colorectal cancer (CRC) is the third most common malignancy and one of the best examples of a tumor tightly associated with prolonged inflammation^1^. One outstanding question about CRC is how both environmental and intracellular factors contribute to the development and pathogenic mechanisms of the disease^2, 3^. It is well known that there is higher risk of CRC in patients with inflammatory bowel disease (IBD), including ulcerative colitis and Crohn’s disease, compared to the general population^4–6^. Although the exact etiology and detailed mechanisms underlying CRC remain unclear, CRC is thought to result from breakdown of the epithelial barrier, followed by inflammatory reactions often linked to the microbial response, even to the bacterial strains populated that have a symbiotic relationship with the host^7, 8^. Currently, several inflammatory cells have been implicated in the pathophysiology of IBD, including mast cells^9^, macrophages^10^, neutrophils^11, 12^, and CD103^+^ DCs^13–15^, as well as lymphoid cells^16^. These inflammatory cells contribute to the transition of colitis to CRC by releasing vasoactive substances or cytokines^17^. In IBD, several inflammatory cytokines, such as interleukin-1β (IL-1β), interleukin 6 (IL-6) and TNF-α, mediate the progression of colitis and CAC^18–20^. However, the regulatory network composed of inflammatory cells and inflammatory factors are not entirely clear.

Both IL-1β and IL-18 are two strong proinflammatory cytokines that can also modulate the activation and effector functions of neutrophils^21–24^, macrophages^25, 26^, mast cells^27, 28^ and dendritic cells^29^ in chronic inflammation. In addition, protein complexes called inflammasome can activate intracellular caspase-1 autocatalytically, which cleaves the inactive precursors of both IL-1β and IL-18 into bioactive cytokines. Elevated levels of IL-1β have been found in the intestines of patients suffering from IBD, which indicates that IL-1β is related to the progression of IBD^30^. It should be noted that the innate immune cells mentioned above are not the only source of IL-1β and IL-18, as epithelial cells can also produce IL-1β and IL-18^31^.

Generally, two identical immunoglobulin (Ig) light chains (IgLCs) are linked to two identical Ig heavy chains by disulfide bonds to form tetrameric Ig. However, many more free IgLCs (FLCs) exist as monomers (molecular weight 22–27 kDa), and as covalently or noncovalently bound dimers (44–55 kDa) or polymers^32, 33^. Unlike the covalent dimerization of FLC through the interchain disulfide bond between the C-terminal cysteines, the noncovalent dimerization of FLC is governed by the amino acid residue present in the framework region of the variable domain through hydrogen bond and hydrophobic bond^34–36^. So the noncovalently bound dimer FLC cannot be reduced to monomers by reducers, like the DTT (DL-Dithiothreitol). FLCs are present in normal serum and can also be detected in urine and cerebrospinal fluid^37, 38^. Historically, excess FLC was considered to be a bystander without any function. In recent years, growing evidence has indicated that FLC is significantly linked to the progression and severity of inflammatory diseases, and both covalent and noncovalent dimerization of FLC are closely associated with the formation of fibrillar deposit in many diseases^35, 39–41^. Early clinical reports regarded FLC as biomarkers of many inflammatory diseases, such as autoimmune disease, diabetes mellitus and CNS inflammation^42, 43^. Furthermore, elevated FLC depositing in the extracellular matrix often resulted in inflammation and tissue injury; two examples of this phenomenon include amyloid light chain amyloidosis (AL-AM) and light chain deposition disease (LCDD)^44–46^. Recently, it was found that FLC might be involved in the induction of hypersensitivity reaction by activating mast cells and promoting their degranulation via unknown receptors^47, 48^. In addition, FLC binds to neutrophils and prompts the release of CXCL8, which blocks the apoptosis of neutrophils and contributes to severe chronic inflammation^49^. Moreover, in a large cohort of breast cancer patients, FLC expression was also shown to be associated with basal-like cancers with aggressive phenotypes^50^.

Rijnierse *et al*. found that FLC levels were increased in the serum of patients with IBD. Moreover, increased levels of FLC were detected in colon specimens from patients with IBD^48^. Importantly, it has been reported that FLC induces hypersensitivity-like IBD with mechanism unknown. In this study, two mouse models of DSS-induced colitis and AOM/DSS-induced CAC were established, and peptide F991, a functional inhibitor of FLC, was used to block FLC activity. We found that F991 significantly attenuated colitis progression and carcinogenesis and greatly reduced the infiltration of inflammatory cells in both colitis and CAC tissues, including activated neutrophils. Furthermore, we showed that F991 blocked inflammasome activation by inhibiting the cleavage of caspase-1 and the activation of IL-1β and IL-18.

## Results

### FLC was evaluated in DSS-induced colitis tissue

**Figure 1:**
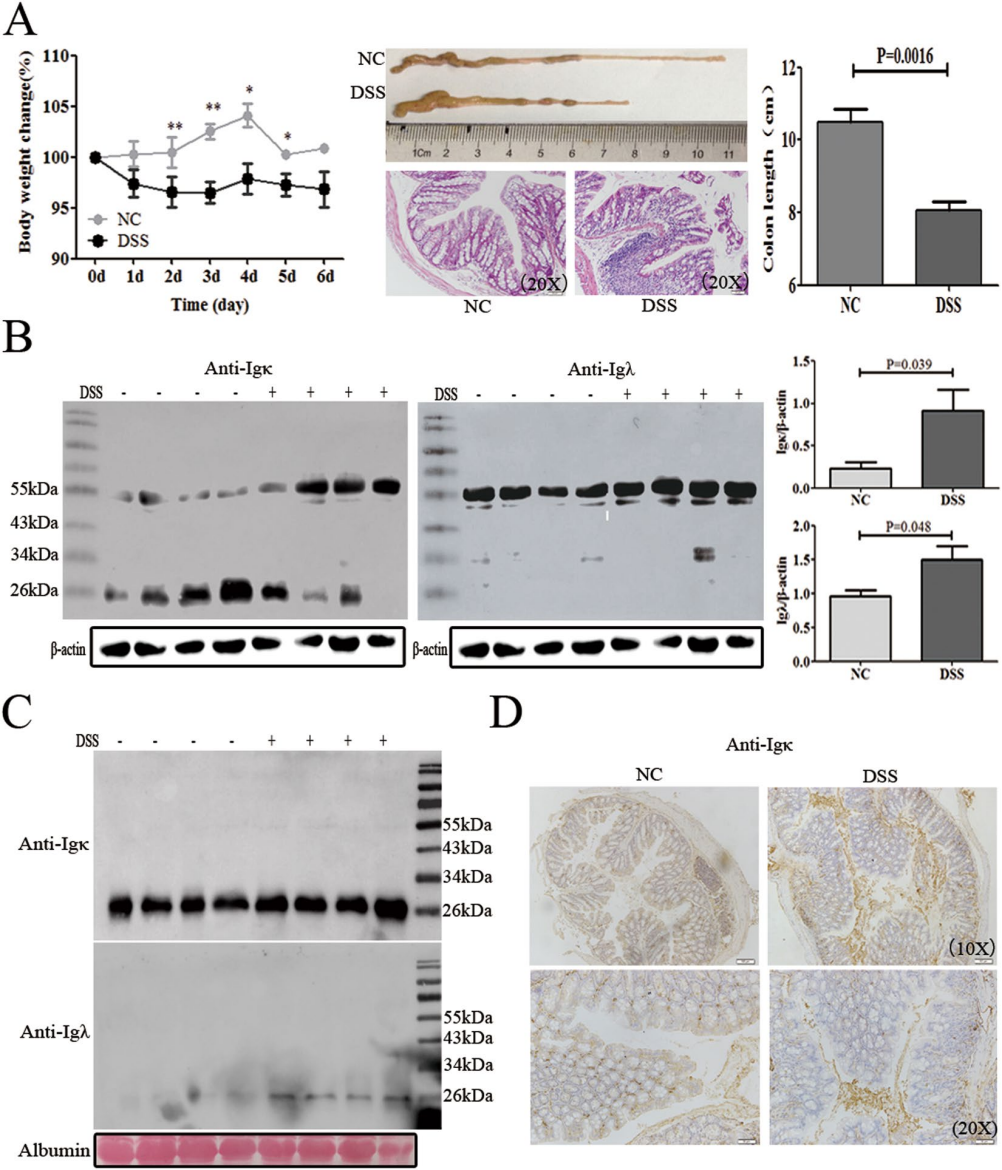
FLC was evaluated in the DSS-induced colitis tissues. Mice were treated with 4% DSS in their drinking water for 6 days to induce acute colitis. The mice were sacrificed on 6^th^ day to detect the level of FLC in the serum and colon tissues. (**A**) The body weight and colon length are shown. Scale bars: 50 μM. The results are shown as the mean ± SD of 4 mice per group. *ns: no statistical significance*, *P < *0.05*, **P < *0.01* compared with the control group. (**B**) Igκ and Igλ in the hydrophobic component of colon tissue were quantified by reduced western blot. (**C**) Igκ and Igλ in the serum were detected by reduced western blot. (**D**) The expression of Igκ is shown in the colon tissue with immunohistochemistry. Scale bars: 100 μM and 50 μM. *NC*: normal control mice, *DSS*: mice challenged with DSS. Data shown are representative of 3 experiments.

To establish the DSS-induced colitis model, mice were challenged with 4% DSS in water. After 6 days, the mice exhibited weight loss, rectal bleeding and diarrhea, characteristics of severe colonic inflammation (Fig. 1A). We then compared the FLC levels of both κ and λ chains in colon tissue between DSS-induced colitis and normal mice. In the colon tissue, the data of reduced western blot showed that both Igκ and Igλ were mainly present as dimer in free form, which was different from the IgLC in intact Ig that should be reduced to monomer (25–29 kDa), but not the dimer. Moreover, the dimeric FLC of both κ and λ chains were significantly increased in the colon tissue of DSS-induced colitis mice (P = 0.039 and P = 0.048) (Fig. 1B). The detection of Igκ and Igλ by non-reduced western blot in colon tissue were also shown as free dimeric form (Supplementary Fig. S1). However, Igκ and Igλ in the serum were only existed as 26 kDa under reduced or non-reduced condition, but not 55 kDa (Fig. 1C and Supplementary Fig. S1), which suggested that the dimeric noncovalent FLC mainly deposited in tissue with no or few in serum. Immunohistochemistry also revealed increasing positive staining for FLC in the extracellular space of the inflammatory tissues from DSS-induced colitis mice (Fig. 1D). These results suggested that FLC might be involved in pathological process of DSS-induced colitis.

### F991 attenuated the inflammation progression in DSS-induced colitis

**Figure 2:**
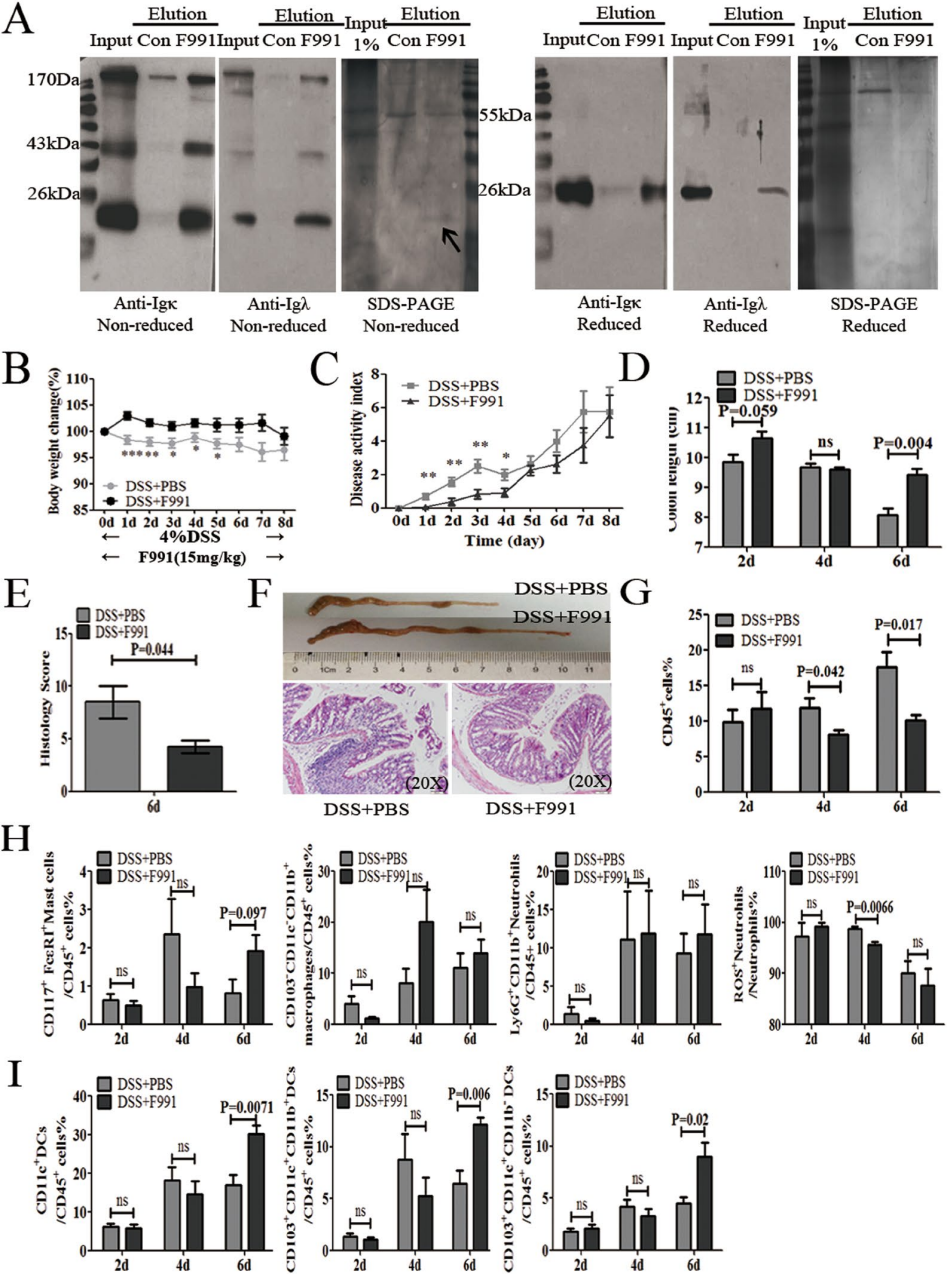
F991 attenuated the inflammatory progression of DSS-induced colitis (**A**) Colon lysates from WT mice were extracted by TSD lysis buffer, and then purified by the F991-coupled column, using the control peptide-coupled column as the control column. Input, elution were analyzed by western blot and SDS-PAGE under non-reducing condition and reducing condition with anti-Igκ and anti-Igλ antibody. One representative experiment of two is displayed. (**B**) and (**C**) The body weight and disease activity index of the mice were measured when the mice were treated with 4% DSS in their drinking water for 8 days to induce acute colitis, and F991 (15 mg/kg) was administered daily via i.p. injection. In addition, the same volume of PBS was administered through i.p. in F991-vehicle mice. (**D**) The length of the colon was measured when the mice were sacrificed on the 2^nd^, 4^th^ or 6^th^ day. (**E**) Histology score of the severity of inflammatory lesions was evaluated after treatment of F991 when the mice were sacrificed on 6^th^ day. (**F**) The length of the colon and the (**H** & **E**) staining are shown after F991 treatment. Scale bars: 50 μM. (**G**–**I**) All leukocytes (CD45^+^ cells), mast cells, macrophages, neutrophils and subpopulations of DCs in the LP, were isolated and counted on the 2^nd^, 4^th^ and 6^th^ day by flow cytometric assay. The results (**B**–**I**) are shown as the mean ± SD of 4 mice per group at each time point. *ns: no statistical significance*, *P < *0.05*, **P < *0.01*, ***P < *0.001* compared with group DSS + PBS. *Con:* control peptide, *DSS* + *PBS*: mice challenged with DSS and injected with PBS, *DSS* + *F991*: mice challenged with DSS and injected with F991. One representative experiment of three is displayed.

F991 has been identified as an FLC inhibitor^47–51^. In this study, we used an F991-affinity column to confirm that F991 preferentially bonded with FLC, than other proteins (Fig. 2A). To determine whether FLC activity was involved in DSS-induced colitis, F991 was intraperitoneally injected into DSS-induced colitis mice to block FLC activity. F991 significantly reduced weight loss, rectal bleeding and diarrhea in the DSS-induced colitis mice. Correspondingly, the disease activity index, a clinical parameter that reflects the severity of weight loss, rectal bleeding and stool consistency, was reduced in F991-treated mice (Fig. 2B,C). Moreover, it was found that F991 alleviated shortening of the colon caused by DSS treatment (P = 0.004), and the H&E staining also revealed that F991 significantly reduced the severity (histological score) and extent of inflammatory lesions (P = 0.044) (Fig. 2D–F).

Oral DSS administration physically disrupts the mucosal barrier and exposes the lamina propria (LP) immune cells to lumen bacterial products, resulting in colitis; the severity of colitis depends upon the concentration of DSS used and the number of consecutive treatment days^52, 53^. We observed infiltration of several inflammatory cells into the LP on the 2^nd^, 4^th^ and 6^th^ treatment days, when the mice were challenged with 4% DSS accompanied by 15 mg/kg F991. Inflammatory cells isolated by enzymatic digestion from the LP were analyzed by FACS. F991 significantly decreased the infiltration of total inflammatory cells (CD45^+^) (P = 0.042 on 4^th^ day, P = 0.017 on 6^th^ day) (Fig. 2G). As expected, the percentage of active neutrophils (CD45^+^ Ly6G^+^ CD11b^+^ ROS^+^) on the 4^th^ day was significantly reduced (P = 0.0066), and changes in the percentage of mast cells (CD45^+^CD117^+^FcεRI^+^) and macrophages (CD45^+^CD103^−^CD11c^−^CD11b^+^) were not significant (Fig. 2H). Notably, CD103^+^ dendritic cells (DCs), the main DC population in the colonic LP, including the main two population of DCs, CD103^+^CD11b^+^CD11c^+^and CD103^+^CD11b^−^ CD11c^+^, were significantly increased by F991 treatment in the DSS-induced colitis mice on the 6^th^ day (P = 0.0071, P = 0.0069 and P = 0.02, respectively) (Fig. 2I).

### F991 relieved inflammasome activation in the DSS-induced colitis

**Figure 3:**
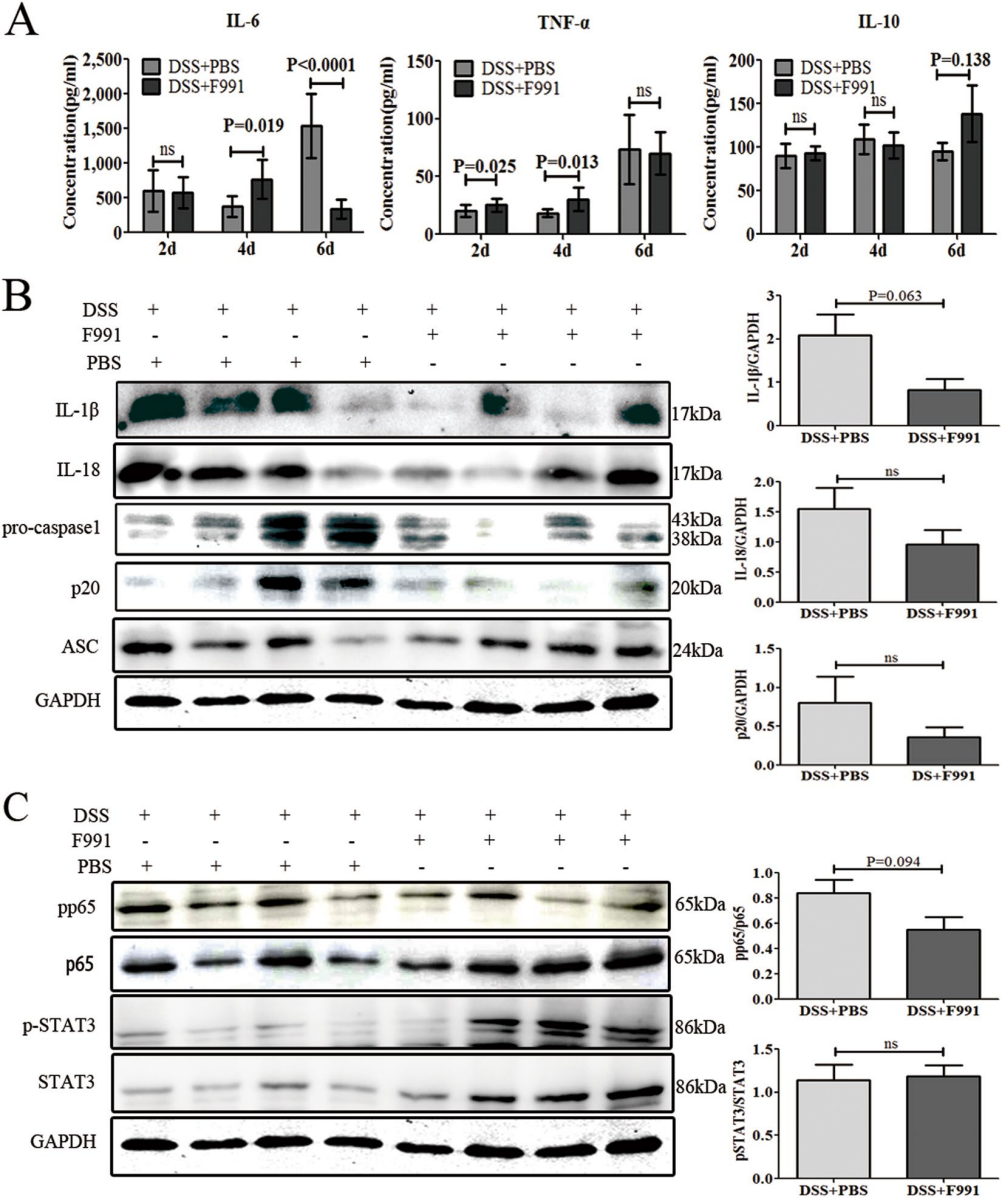
F991 relieved inflammasome activation in the inflammatory tissue of DSS-induced colitis mice. (**A**) The level of IL-6, TNFα, IL-10 in the cultured supernatant of colonic tissue on the 2^nd^, 4^th^ and 6^th^ day were examined by ELISA. The results are shown as the mean ± SD of 4 mice per group at each time point. *ns: no statistical significance*, *P < *0.05*, **P < *0.01*, ***P < *0.001* compared with group DSS + PBS. (**B**) The level of active IL-1β, IL-18, pro-caspase-1, cleaved caspase-1 (p20) and ASC in the colonic tissues on the 6^th^ day were determined by western blot. (**C**) The level of phosphorylated p65 (p-p65), and phosphorylated-STAT3 (p-STAT3) in the colonic tissues on the 6^th^ day were analyzed by western blot. *DSS* + *PBS*: mice challenged with DSS and injected with PBS, *DSS* + *F991*: mice challenged with DSS and injected with F991. One representative experiment of three is displayed.

To investigate how F991 reduced DSS-induced colitis, we used ELISA to analyze the levels of proinflammatory cytokines, including IL-6 and TNF-*α*, and the levels of the anti-inflammatory cytokine IL-10 in cultured supernatant from DSS-colitis tissues after F991 treatment on the 2^nd^, 4^th^, 6^th^ days. We found that IL-6 levels were unchanged on the 2^nd^ day, increased on the 4^th^ day, and significantly reduced by nearly five times (P < 0.0001) on the 6^th^ day. Additionally, TNF-α levels temporarily increased on the 2^nd^ and 4^th^ days but were unchanged on the 6^th^ day. As expected, IL-10 was increased by F991 treatment, but was not statistically significant (P = 0.138 on 6^th^ day) (Fig. 3A).

Active IL-1β and IL-18, which are derived from pro-IL-1β and pro-IL-18 depending on the protease activity of caspase-1 in the inflammasome, were also considered to be involved in the pathogenesis of IBD. Notably, both active IL-1β and IL-18 were markedly decreased on the 6^th^ day (P = 0.063 and P = 0.217). As expected, active caspase-1 (p20) was also reduced by F991 treatment on the 6^th^ day without statistical significance(Fig. 3B). These results suggested that FLC could be involved in the activation of the inflammasome in DSS-induced colitis. However, ASC, another member of the inflammasome, showed no change on the 6^th^ day (Fig. 3B). We also evaluated inflammasome activity after F991 treatment on 4^th^ day, but the levels of IL-1β, IL-18 and the p20 subunit of caspase-1 were too low to be detected (Supplementary Fig. S2).

In addition, we also analyzed the inhibitory effect of F991 on the activation of NF-κB, which is essential for IL-6 production, as well as IL-6-mediated phosphorylation of STAT3. We found that phosphorylated p65 decreased on the 4^th^ and 6^th^ day, but was not statistically significant (P = 0.221 and P = 0.094). Unexpectedly, phosphorylated STAT3 was decreased on the 4^th^ day and did not change by F991 treatment on the 6^th^ day, which was incongruent with the decreased IL-6 levels at this time point as described above (Fig. 3C).

### F991 suppressed tumorigenesis by inhibiting FLC activation in the AOM/DSS-induced CAC model

**Figure 4:**
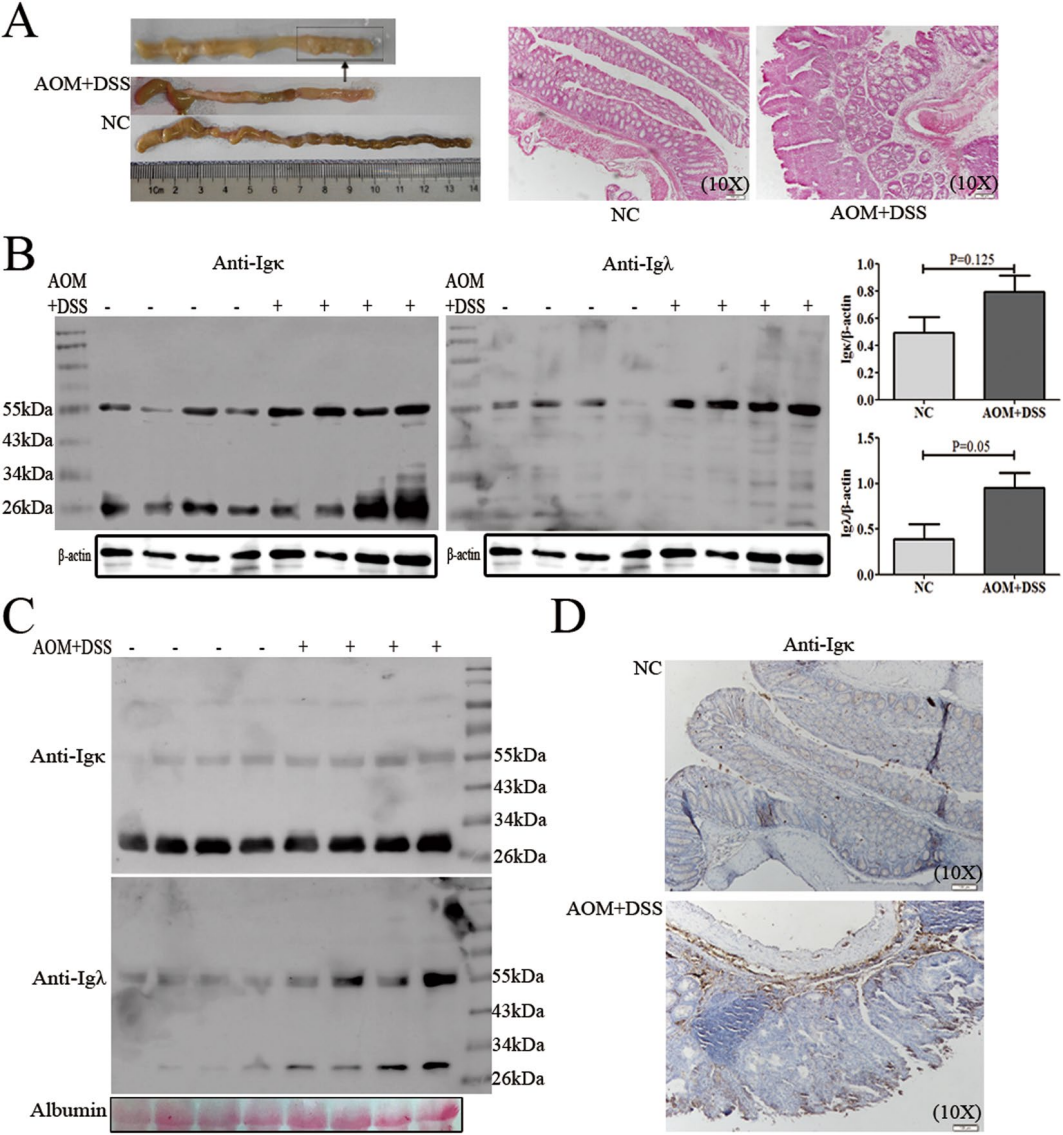
FLC was evaluated in the AOM/DSS-induced colitis-associated colorectal cancer tissues. Mice were injected i.p. with a single dose (10 mg/kg) of AOM, and then mice were given 3 cycles of 2.5% DSS administered in the drinking water for 7 days, followed by 14 days of regular water. Mice were sacrificed on day 91 after CAC induction. (**A**) A number of visible tumors on day 91 is shown. Scale bars: 100 μM. (**B**) Igκ and Igλ in the hydrophobic component of colon tissue were quantified by reduced western blot. (**C**) Igκ and Igλ in the serum were detected by reduced western blot. (**D**) The Igκ staining in the colon or tumor tissues was performed with immunohistochemistry. Scale bars: 100 μM. *NC*: normal control mice, *AOM* + *DSS*: AOM/DSS-induced CAC mice. One representative experiment of two is displayed.

To evaluate whether FLCs were increased during CAC tumorigenesis, we used AOM and DSS to induce CAC (Fig. 4A). After treatment for 91 days, the FLC levels of both κ and λ chains were evaluated in CAC tissue, the result showed that only the dimeric (55 kDa) FLC of either κ or λ chains under reduced condition, but not the monomeric IgLC, was significantly elevated (P = 0.125 and P = 0.05) (Fig. 4B and Supplementary Fig. S3). Besides the increasing level of dimeric Igλ in the serum, we interestingly found that Igκ and Igλ monomer existed as 29 kDa under reduced or non-reduced condition, but not the conventional 26 kDa (Fig. 4C and Supplementary Fig. S3), which might be N-glycosylated IgLC (our unpublished data). Immunohistochemistry in AOM/DSS-induced CAC mice also revealed stronger positive staining for FLC in cells surrounded with carcinomas(Fig. 4D). But whether they are mesenchymal cells or infiltrated immune cells remained unknown, which need further investigation. These results suggested that FLC might also be involved in the progression of DSS-induced colitis to CAC.

**Figure 5:**
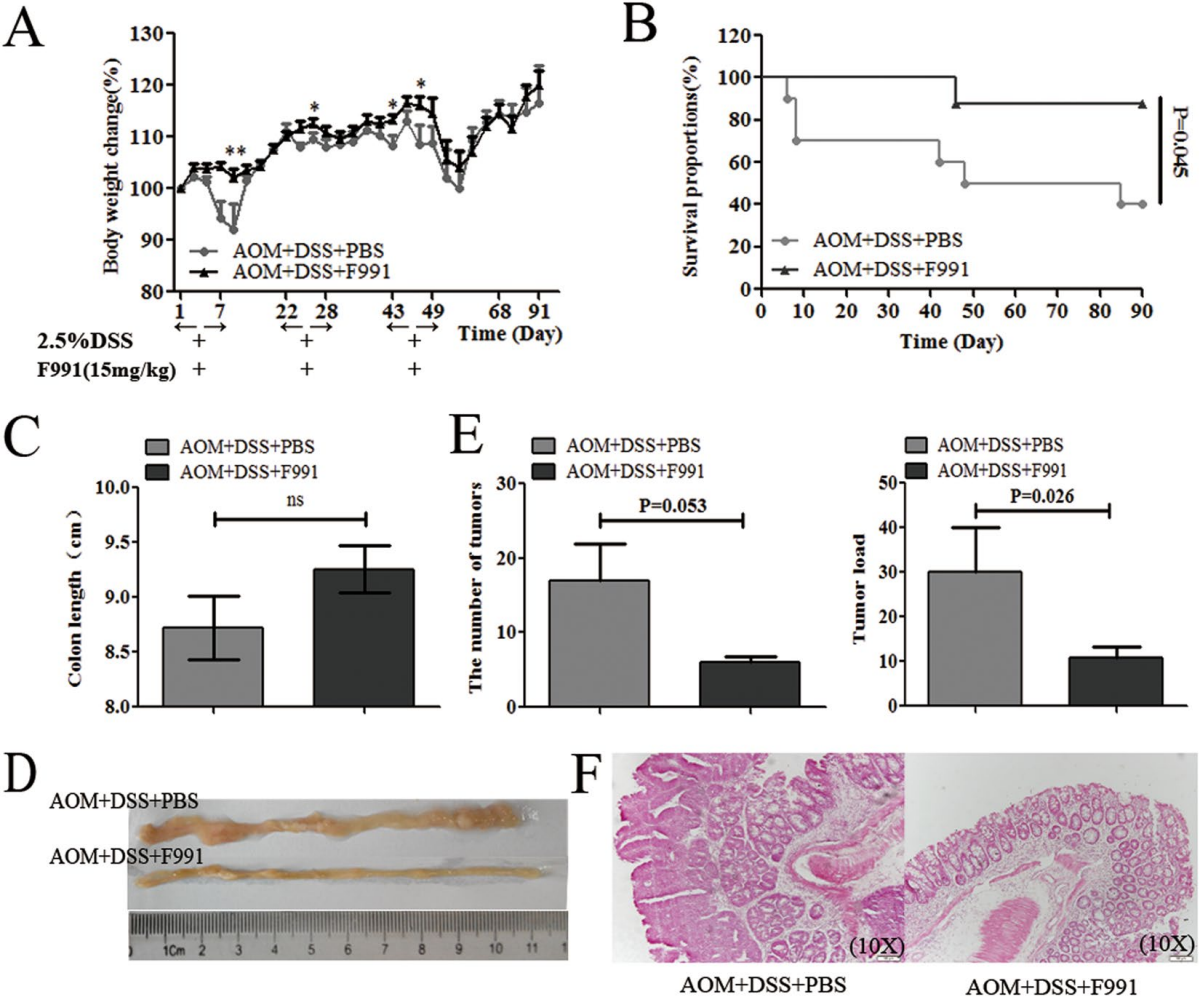
F991 suppressed tumorigenesis and improved the survival rate in the AOM/DSS-induced CAC model. Mice were injected i.p. with a single dose (10 mg/kg) of AOM, and then mice were given 3 cycles of 2.5% DSS administered in the drinking water for 7 days, followed by 14 days of regular water. F991(15 mg/kg) was administered through i.p. injection daily in conjunction with the DSS treatment cycles, and the same volume of PBS was administered through i.p. in F991-vehicle mice. Mice were sacrificed on day 91 after CAC induction. (**A**) Body weight and (**B**) the survival rate of the AOM/DSS-induced CAC mouse model were monitored. (**C**) The colon length was measured after the mice were sacrificed on day 91. (**D**) Colonic tumor formation is shown. (**E**) Tumor number was measured and the tumor load was determined by totaling the diameter of all tumors for a given animal. (**F**) Colon tissues were stained with H&E. Scale bars: 100 μM. The results (**A**–**E**) are shown as the mean ± SD of 10 mice per group. *ns: no statistical significance*, *P < *0.05* and **P < *0.01*, compared with group AOM + DSS + PBS. *AOM* + *DSS* + *PBS*: AOM/DSS-induced CAC mice and injected with PBS, *AOM* + *DSS* + *F991*: AOM/DSS-induced CAC mice and injected with F991. One representative experiment of two is displayed.

To evaluate whether F991 could suppress CAC tumorigenesis, we injected 15 mg/kg F991 during each DSS challenge. Compared to controls without F991, F991 effectively eliminated the body weight loss induced by DSS (Fig. 5A). Moreover, F991 significantly reduced mortality rates, particularly in the early stage (P = 0.045) (Fig. 5B). After visible tumor formation in CAC, we compared the colon length, tumor size and the quantity of tumors between the two groups. We found that the average colon appeared to be longer in the F991-treated mice, but the difference between the groups was not statistically significant due to the small number of surviving mice in the untreated control group (Fig. 5C). However, tumor load (including the number and the size of tumors) was significantly decreased by F991 treatment (P = 0.053, P = 0.026) (Fig. 5D–F).

### F991 inhibited inflammasome activation and inflammatory cell infiltration in the AOM/DSS-induced CAC model

**Figure 6:**
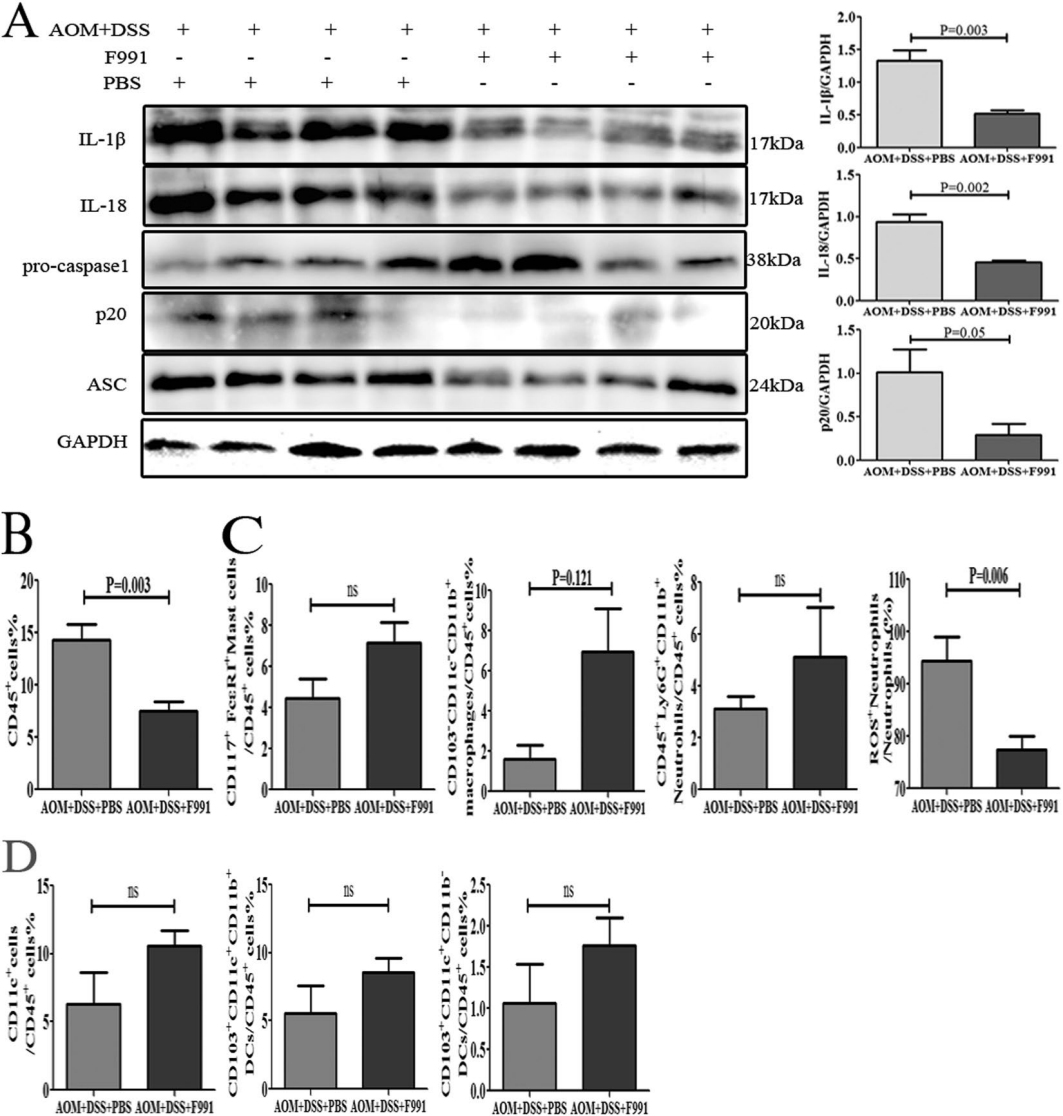
F991 inhibited inflammasome activation and inflammatory cell infiltration in the AOM/DSS-induced CAC model. (**A**) Levels of active IL-1β, IL-18, pro-caspase-1, cleaved caspase-1 (p20) and ASC in the colonic tissues were determined by western blot. Data were from 4 mice were shown. (**B**) CD45^+^ cell, (**C**) mast cells, macrophages, neutrophils and (**D**) subpopulations of DCs were detected using flow cytometry. The results (**B**–**D**) are shown as the mean ± SD of 10 mice per group. *ns: no statistical significance*, *P < *0.05* and **P < *0.01*, compared with the group AOM + DSS + PBS. *AOM* + *DSS* + *PBS*: AOM/DSS-induced CAC mice and injected with PBS, *AOM* + *DSS* + *F991*: AOM/DSS-induced CAC mice and injected with F991. One representative experiment of two is displayed.

We investigated whether F991 treatment could also reduce activation of the inflammasome in the CAC model. Our results clearly demonstrated that F991 suppressed active IL-1β, IL-18 and active caspase-1 (p20) in the AOM/DSS-induced CAC model (P = 0.003, P = 0.002 and P = 0.05) (Fig. 6A). Subsequently, we analyzed inflammatory cell infiltrate in the CAC tissues after F991 treatment, and found that the percentage of CD45^+^ leukocytes was markedly reduced (P = 0.003) (Fig. 6B), especially the ROS^+^ neutrophils (P = 0.006) (Fig. 6C). However, there were no significant changes in the percentage of mast cells, macrophages, and CD103^+^DCs (Fig. 6C,D).

## Discussion

Ulcerative colitis (UC), a common form of IBD characterized by chronic remitting and relapsing inflammation, usually affects the large intestine and increases the risk of colitis-associated carcinoma. Moreover, both DSS-induced colitis and AOM/DSS-induced colorectal cancer models share immunological and histopathological features with UC patients^53, 54^. In this study, for the first time, we found that FLC could promote colitis, especially in the AOM/DSS-induced CAC mice, by activating the inflammasome.

Growing evidence indicates that FLC is involved in the pathogenesis of many inflammatory diseases, such as autoimmune diseases and hypersensitivity. A previous study reported that FLC could promote hypersensitivity-like IBD^48^, but it was unclear whether FLC was involved in colitis. In this study, we used the DSS-induced colitis mouse model, and found that FLC of either κ or λ chains was significantly enhanced in the tissues of DSS-induced colitis mice. Importantly, F991, the FLC inhibitor^47–49, 51^, significantly suppressed the progression of DSS-induced colitis.

It was also found that aggressive tumors, which expressed higher levels of FLC, could also be reduced by F991 treatment^50^. This finding suggested that FLC might promote tumor-associated inflammation, possibly even the transition from inflammation to cancer. Therefore, we also used the AOM/ DSS-induced CAC mouse model in this study. Similarly, we found that FLC of either κ or λ chains was significantly enhanced in the tissue of AOM/ DSS-induced CAC mice and that F991 inhibited tumor formation, and prolonged survival time. Our results are the first to reveal that FLC is involved in tumor-associated inflammation and the transformation of inflammation to cancer.

FLC promoted activation of neutrophils in both models of DSS-induced colitis and AOM/ DSS-induced CAC. According to current research, FLC promotes degranulation of mast cells in hypersensitivity^47, 51^ or promotes the survival of neutrophils in inflammatory diseases^49, 55^. Moreover, many studies have reported that neutrophils extracellular traps (NETs), depending on the formation of reactive oxygen species (ROS) within intracellular granules^56^, are potential proinflammatory factors^57–59^. In this study, we first analyzed the percentage of inflammatory cells, including mast cells, neutrophils, CD103^+^ dendritic cells, macrophages and lymphocytes, after F991 treatment. In the DSS-induced colitis model, we found that neutrophils, especially ROS^+^ neutrophils were significantly reduced. Interestingly, the CD103^+^ dendritic cells, which were considered to play a protective role in colonic LP tolerance to commensal bacterias and food antigens under physiological conditions^60, 61^, were markedly increased. In the AOM/ DSS-induced CAC model, the ROS^+^ neutrophils, but not other myeloid cells, were significantly reduced by F991. These results were similar to previous findings^47, 48^.

FLC appeared as a dimer in both models of DSS-induced colitis and AOM/ DSS-induced CAC. Usually, FLC exists as a monomer (25–29 kDa), dimer (45–55 kDa), oligomer or polymer. Generally, the monomeric form is usually present in blood, and the dimeric, oligomeric or polymeric forms are limited to local deposits^62^. Changes in the structure and rising levels of dimeric FLC occur in many relevant diseases, such as AL-amyloidosis, multiple myeloma (MM), and multiple sclerosis (MS), and are associated with the pathogenesis of these diseases^36^. In this study, we found the FLC of either κ or λ chains increased in the 55 kDa dimeric form in colitis or AOM/ DSS-induced CAC. However, the level of dimeric FLC in the colon were unchanged after F991 treatment (Supplementary Figs S4 and S5), which confirmed that F991 blocked FLC and did not interfere with its production and dimerization^63, 64^.

FLCs were known to induce activation of the inflammasome, but the proinflammatory molecular mechanism of FLC remained unclear. To address the mechanism of dimeric FLC, we further analyzed some proinflammatory cytokines, such as IL-6, TNF-α, IL-1β and IL-18. We found that the levels of active IL-1β and IL-18, which are induced by the inflammasome, but not IL-6 and TNF-α, were associated with increased FLC and inflammatory damage in colitis tissues or tumor formation in AOM/ DSS-induced CAC. More importantly, F991 markedly reduced activated IL-1β and IL-18, as well as activated caspase-1. Our results suggested that the proinflammatory effect of dimeric FLC is mediated by the inflammasome. Moreover, blocking IL-1β and IL-18^65–67^ or inhibiting caspase-1^68, 69^ effectively protected mice against colitis. In addition, IL-1β and IL-18 were previously reported to delay neutrophil apoptosis and prolong their release of potentially harmful enzymes^21, 70^, which was similar to the effect of FLC on neutrophils.

However, in this study, there were three questions. First, the CD103^+^ DCs are the main population of DCs in colonic LP. However, it remained unclear whether the CD103^+^ DCs contribute to the pathogenesis of IBD and CAC. In this study, we found that CD103^+^ dendritic cells (DCs), including the main two populations of DCs, CD103^+^CD11b^+^CD11c^+^and CD103^+^CD11b^−^ CD11c^+^, were significantly increased by F991 in the DSS-induced colitis model, but the mechanism needs to be further investigated. Second, while the size of monomer IgLC (including κ or λ chains) was 25 kDa in the blood of DSS-induced colitis mice, the size of monomer IgLC in blood of AOM/ DSS-induced CAC mice was 29 kDa, which was identified as N-glycosylated (data not shown). The significance of FLC N-glycosylation needs to be addressed by further research. Thirdly, though Igλ expression was extremely low under physical condition, elevating Igλ usually linked with infection^71, 72^ indicating Igλ was inducible. So inducing expression of Igλ in our animal models might result from the disruption the mucosal barrier and exposition LP cells to lumen bacteria by oral DSS administration. But the exact precise pathway need to be further studied.

Our results are the first to demonstrate that elevated FLC promotes inflammation-related carcinogenesis via activation of the inflammasome. We also showed that blocking FLC with F991 alleviates inflammatory damage and tumor load and improves survival rates. Therefore, our data may inform the use of FLC as a biomarker for inflammatory bowel disease diagnosis in patients, and the use of F991 as a potential therapeutic option.

## Materials and Methods

### Ethical Considerations

Every effort was made to minimize the number of animals used as well as their suffering with the approval of Institutional Animal Care and Use Committee of China in accordance with the Guide for the Care and Use of Laboratory Animals (NIH, USA).

### Mice and induction of colitis

Balb/C mice, 6–8 wk old, were purchased from Beijing Hua Fukang Bioscience Co., Ltd, China. Mice were housed at the Department of Laboratory Animal Science of Peking University with free access to pellet food and water in plastic cages at 21 ± 2 °C and kept on a 12 h light/dark cycle. Study procedures were performed in accordance with the guidelines of the People’s Republic of China Ministry of Health and approved by Peking University Health Science Center.

DSS (MW: 36,000–50,000, 160110, MP Biomedicals) was dissolved in drinking water at a dilution of 4% (w/v) and administered for 7 days to induce acute colitis in wild type (WT) mice. F991 (AHWSGHCCL, synthesized by LifeTein LLC, Beijing, China) (15 mg/kg) diluting in PBS was administered through i.p. (intraperitoneal) injection daily at the indicated dose for 7 days. In addition, the same volume of PBS was administered through i.p. injection in F991-vehicle mice. To ensure the same average weight of groups, mice were randomly divided into three groups, which were consecutively killed every two days (day 2, day 4, day 6).

A clinical score ranging from 0 to 10 was used to evaluate the severity of the colitis. Body weight, stool consistency, and the presence of gross blood in feces and in the anus were observed every day. The disease activity index was calculated by assigning well-established and validated scores. Briefly, the following parameters were used for calculation: a) loss in body weight (0 = no loss; 1 = 5–10%; 2 = 10–15%; 3 = 15–20%; 4 =  > 20%), b) diarrhea (0 points = normal, 2 points = soft stools, 3 points = loose stools, 4 points = watery diarrhea); c) hematochezia (0 points = no bleeding, 2 points = slight bleeding, 4 points = gross bleeding). The entire colon and rectum were processed for histopathological examination and further experiments. One half of the distal colon was taken for infiltrating immune cells analyses and supernatant cytokine analyses. The other half was fixed in 10% neutral buffered formalin for 24 h and transferred to 70% ethanol for subsequent paraffin embedding and histological analysis. The clinical course of the disease was followed daily by measurement of body weight and monitoring for signs of rectal bleeding or diarrhea.

### Induction and treatment of colitis-associated cancer and colitis

To induce CAC, mice were injected i.p. with a single dose (10 mg/kg) of azoxymethane (AOM, A5486, Sigma-Aldrich) and kept on regular diet with free access to water for 7 days. Afterwards, the mice received water with 2.5% DSS for 7 days followed by regular water for 14 days and were then subjected to two more DSS treatment cycles. F991(15 mg/kg) was administered through i.p. injection daily in conjunction with the DSS treatment cycles. In addition, the same volume of PBS was administered through i.p. in F991-vehicle mice. Mice were sacrificed on day 91 after CAC induction.

Macroscopic tumors were counted and measured with a caliper. The entire colon and rectum were processed for histopathological examination and further experiments. The clinical course of the disease was monitored every other day by measurement of body weight during the period of DSS administration and every three days in the remaining periods.

### Preparation of F991-coupled sepharose column

Affinity purified F991 was coupled to CNBr-activated sepharose 4B beads (17-0430-01, GE) for preparation of the affinity column. Purified F991 or control peptide (5 mg) was diluted in 6 ml coupling buffer (0.1 M NaHCO_3_, 0.5 M NaCl, pH 8.3). Sepharose beads were then introduced to 1 mM HCl for 30 min and washed with coupling buffer. The F991 or control peptide (EGFRLSPGLG, synthesized by LifeTein LLC, Beijing, China) was added to resin, incubated overnight at 4 °C and blocked with blocking buffer (100 mM Glycine, pH 8.0) for 2 h at room temperature. F991-coupled sepharose and control peptide-coupled sepharose were transferred to the column and washed (3×) with alternating Tris-HCl buffer (0.1 M Tris-HCl buffer pH 8–9 containing 0.5 M NaCl) and acetate buffer (0.1 M acetate buffer pH 3–4 containing 0.5 M NaCl). Before use, these coupled columns were washed (3×) with PBS buffer (pH 7.2).

### Affinity Purification

Colon lysates of WT mice, extracted by 600 μl TSD lysis buffer (50 mM Tris-HCL, pH 8.1, 1% SDS), were diluted with 18 ml Co-IP buffer (1% NP-40, 150 mM NaCl, 50 mM Tris-Hcl, pH 7.4) loaded on a column and incubated overnight at 4 °C. The bound proteins were eluted using elution buffer (100 mM Glycine, pH 2.4) and neutralized with 0.1 M Tris-HCl (pH 9.0). Then ultrafiltrated and analyzed by non-reduced and reduced SDS-PAGE and western blot.

### Histological analysis, immunohistochemistry

To evaluate pathology, the distal colons were dissected out and fixed using 10% neutral buffered formalin overnight before H&E staining and analysis by a pathologist using a light microscope (Olympus).

Histological scoring was based on the three following parameters: a) severity of inflammation: 0 = no inflammation; 1 = mild; 2 = moderate; 3 = severe; b) depth of inflammatory involvement: 0 = no inflammation;1 = mucosa; 2 = mucosa and submucosa; 3 = transmural; c) crypt damage: 0 = intact crypts; 1 = one-third loss of the basal crypts; 2 = two-thirds loss of the basal crypts; 3 = entire crypt loss but intact epithelial surface; 4 = entire crypt loss and change of epithelial surface with erosion. The histological score was calculated by adding the three evaluations, with a maximum possible score of 10.

For immunohistochemistry staining, colon tissue sections were deparaffinized by a xylene series, hydrated through a graded ethanol series (100%, 95%, 70%), and washed in PBS. Then, the sections were treated with 2% hydrogen peroxide for 15 min, blocked with 10% rabbit serum for 30 min at room temperature and incubated at 4 °C overnight with specific primary antibodies. After washing three times with PBS, the slides were incubated with anti-goat-IgG-HRP for 20 min at room temperature, stained with DAB (GK500705, Shanghai Gene Company, China) substrate and then counter-stained with hematoxylin. Images were acquired by microscopy.

### Colon cultures and ELISA on supernatants

To perform the ELISA assay on cultured supernatants, colons were excised from mice and colon samples (1-cm in length) were immersed in 500 μL of RPMI 1640 supplemented with penicillin (500 U/mL), and streptomycin (500 μg/mL, 15140122, Gibco). Colons were incubated overnight in a 24-well culture plate at 37 °C, 5% CO_2_. Supernatants were sampled after 36 h and ELISA was performed with murine IL-6, TNFα, and IL-10 Ready-SET-Go Kits (eBioscience) according to the manufacturer’s instructions. The reaction was stopped with 2N H_2_SO_4_ and the absorbance was measured at 450 nm.

### Isolation of immune cells infiltrating in colon

To analyze immune infiltrate in the gut, colons were dissected from mice and epithelial cells were removed by incubating colons for 30 min at 37 °C with shaking in HBSS supplemented with 5 mmol/L EDTA and 5 mmol/L DTT. Colons were further digested in RPMI 1640 containing 1 mg/mL collagenase IV and 300U/ml DNase I (C8160 and D8070, Sigma-Aldrich) for 30 min at 37 °C then filtered with a 74-μm cell strainer. After centrifugation, pelleted cells were aspirated and washed in PBS 2% FBS.

### Flow Cytometry

Cells were prepared as previously described, and stained for 30 min at 4 °C with relevant antibodies. Cells were acquired and analyzed on a Verse flow cytometer (BD Biosciences). All analyzed immune cells were acquired by gate CD45. Local neutrophils were identified as CD11b^+^Ly6G^hi^, mast cells were identified as CD117^+^FcεRI^+^, macrophages infiltrating in the colon were identified as CD103^−^CD11c^−^CD11b^+^ and the two subpopulations of the colon dendritic cells were identified as CD103^+^CD11c^+^CD11b^−^ or CD103^+^CD11c^+^CD11b^+^.The level of ROS in active neutrophils was detected by 0.1 mM DCFH-DA probes (S0033, Beyotime, China) for 30 min at 4 °C per the manufacturer’s instructions.

### Western Blot Assay

Total proteins were extracted from colonic samples using the RIPA lysis buffer (50 mM Tris, pH 7.4, 150 mM NaCl, 1% NP-40, 0.5% sodium deoxycholate, 0.1% SDS) containing protease inhibitors and phosphatase inhibitors (04693116001 and 0490684001, Roche). After centrifugation, the hydrophobic pellet was extracted by TSD lysis buffer (50 mM Tris-HCL, pH 8.1, 1% SDS). The protein concentrations were determined by spectrophotometer using the Bradford protein assay kit (5000205, Bio-Rad Laboratories). After loading equal amounts of protein samples, 10% SDS-PAGE or 12.5% SDS-PAGE was performed. The proteins were then transferred to a nitrocellulose membrane (Millipore). After blocking with Tris-buffered saline containing 0.05% Tween-20 (TBST) and 5% non-fat dry milk for 1 hr, the membrane was incubated with the corresponding primary antibodies at 4 °C overnight, and then washed in TBST. The primary antibodies included goat anti-mouse Igκ (1050-08, Southern Biotec), goat anti-mouse Igλ (1060-08, Southern Biotec), anti-IL-1β (ab 9722, Abcam), anti-IL-18 (sc-7954, Santa Cruz), anti-caspase-1 (14-9832-82, eBioscience), anti-ASC (sc-22514-R, Santa Cruz), anti-p65 (14-6731-81, eBioscience), anti-p-p65 (3033P, CST), anti-STAT3 (9132P, CST), and anti-p-STAT3^Y705^ (9131S, CST). Then, the membrane was incubated with the corresponding horseradish peroxidase-conjugated secondary antibodies for 1 hr, and the proteins were visualized with ECL chemiluminescence (32106, Pierce and RPN2235, GE). β-actin or GAPDH (TA-09 or TA-08, Zhongshan Bio) were used as loading controls, and albumin as the house hold protein in serum was staining by ponceau S.

### Statistical analyses

The densitometry of western blot bands were quantified by ImageJ software. The statistical significance of differences between two groups was determined using a two-tailed unpaired t-test (normally distributed data) and the Mann-Whitney U-test (nonparametric data). All analyses were performed using GraphPad Prism version 5.00 (GraphPad Software). Differences were considered statistically significant at P < 0.05. All data were expressed as the mean ± SEM or SD.

## Electronic supplementary material

SUPPLEMENTARY INFO
