# Supplementary material for: Free immunoglobulin light chain (FLC) promotes murine colitis and colitis-associated colon carcinogenesis by activating the inflammasome
Source: Sci Rep. 2017 Jul 12;7:5165. doi: 10.1038/s41598-017-05468-w (PMC5507933; doi:10.1038/s41598-017-05468-w)
Supplement: Supplementary file 1 — SUPPLEMENTARY INFO [file 41598_2017_5468_MOESM1_ESM.pdf]

Free immunoglobulin light chain (FLC) promotes murine colitis and colitis-associated colon carcinogenesis by activating the inflammasome

Junfan Ma[1,2], Dongyang Jiang[1,3], Xiaoting Gong[1,2], Wenwei Shao[1,2], Zhu zhu[1,2], Weiyan Xu[1,2], Xiaoyan Qiu[1,2, *]

[1.] Department of Immunology, School of Basic Medical Sciences, Peking University Health Science Center, Beijing, 100191, China.

[2]. Key Laboratory of Medical Immunology, Ministry of Health, Beijing, 100191, China

[3]. Department of Cardiology, Cardiovascular Disease Institute, Shanghai Tenth People's Hospital, Tongji University School of Medicine, Shanghai, China

[*]Correspondence and requests for materials should be addressed to X.Q. (email: qiuxy@bjmu.edu.cn)

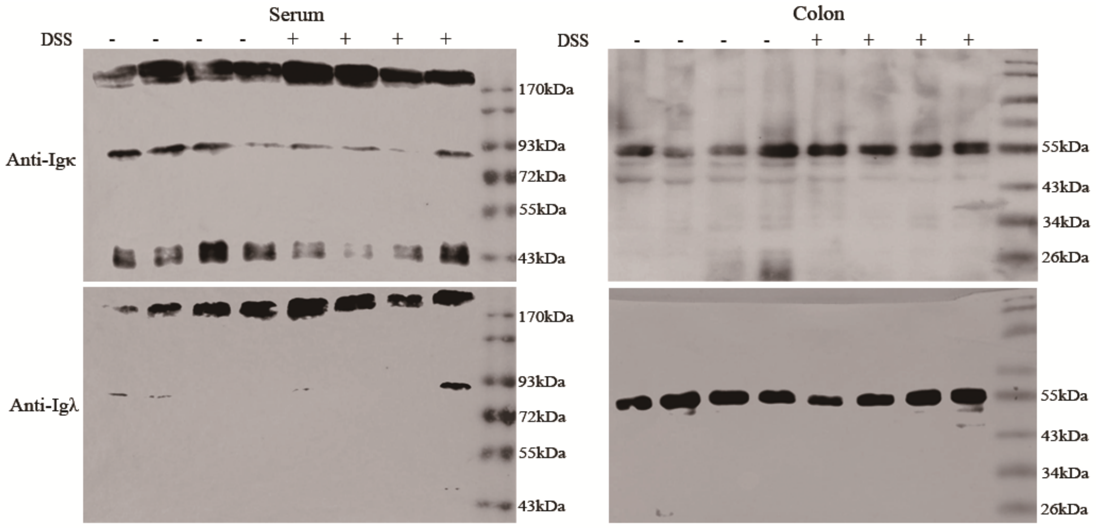

Figure S1: Detection of Igκ and Igλ under non-reduced condition in the DSS-induced colitis tissue and serum. Mice were treated with 4% DSS in their drinking water for 6 days to induce acute colitis and were sacrificed on the 6th day. Igκ and Igλ in (A) colon tissue and (B) serum were detected by non-reduced western blot. There are 4 mice per group. One representative experiment of three is displayed.

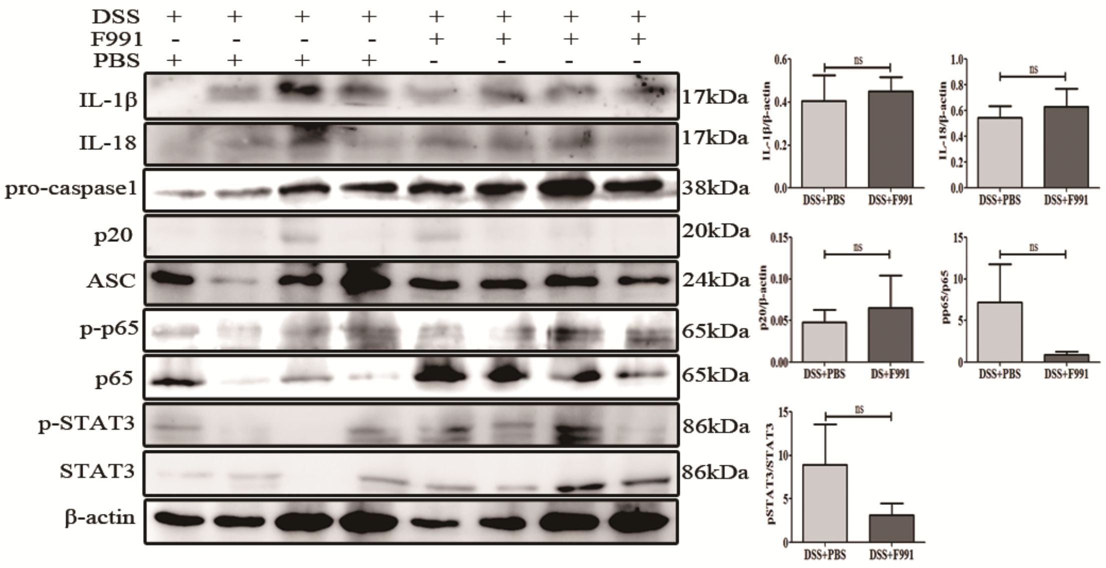

Figure S2: F991 cannot relieved inflammasome activation in the inflammatory tissue of DSS-induced colitis mice on the 4th day. Mice were treated with 4% DSS in their drinking water for 4 days to induce acute colitis, and F991 (15 mg/kg) was administered daily via i.p injection. In addition, the same volume of PBS was administered through i.p.in F991-vehicle mice. The level of active IL-1β, IL-18, pro-caspase-1, cleaved caspase-1 (p20), ASC, phosphorylated p65 (p-p65), and phosphorylated-STAT3 (p-STAT3) in the colonic tissues on the 4th day were

determined by western blot. Data were from 4 mice were shown. *ns: no statistical significance* was showed compared with group DSS+PBS. *DSS+PBS*: mice challenged with DSS and injected with PBS, *DSS+F991*: mice challenged with DSS and injected with F991. One representative experiment of three is displayed.

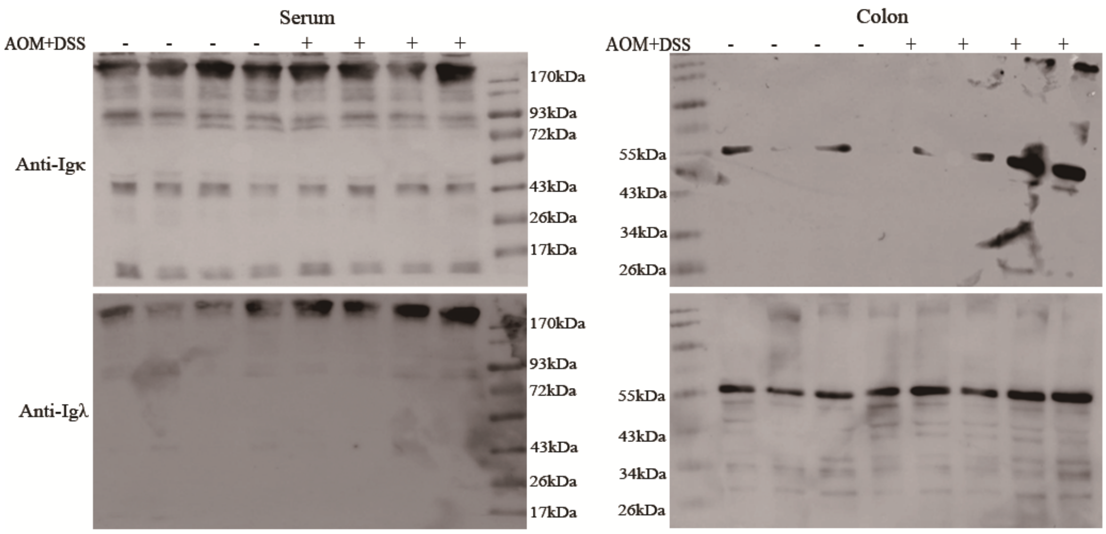

Figure S3: Detection of Igκ and Igλ under non-reduced condition in the AOM/DSS-induced colitis-associated colorectal cancer tissue and serum. Mice were injected i.p. with a single dose (10 mg/kg) of AOM, and then mice were given 3 cycles of 2.5% DSS administered in the drinking water for 7 days, followed by 14 days of regular water. Mice were sacrificed on day 91 after CAC induction. Igκ and Igλ in (A) colon tissue and (B) serum were detected by non-reduced western blot. Data were from 4 mice were shown. One representative experiment of two is displayed.

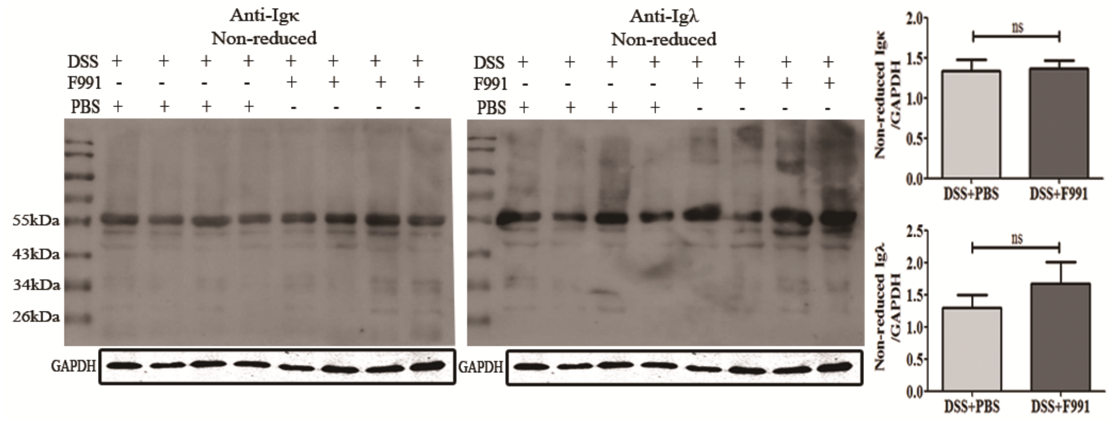

Figure S4: Expression and dimerization of FLC was unchanged after F991 administration in the DSS-induced colitis tissues. Mice were treated with 4% DSS in their drinking water for 6 days to induce acute colitis, and F991 (15 mg/kg) was

administered daily via i.p injection. In addition, the same volume of PBS was administered through i.p.in F991-vehicle mice. The levels of Igκ and Igλ in the colon tissue were quantified by non-reduced western blot. Data were from 4 mice were shown. *ns: no statistical significance* was showed compared with group DSS+PBS. *DSS+PBS*: mice challenged with DSS and injected with PBS, *DSS+F991*: mice challenged with DSS and injected with F991. One representative experiment of three is displayed.

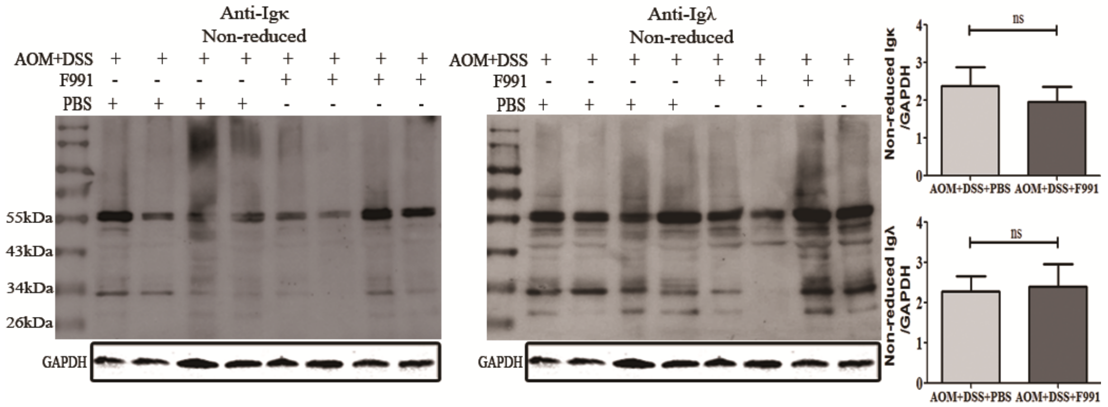

Figure S5: Expression and dimerization of FLC were unchanged after F991 administration in the AOM/DSS-induced CAC model. Mice were injected i.p. with a single dose (10 mg/kg) of AOM, and then mice were given 3 cycles of 2.5% DSS administered in the drinking water for 7 days, followed by 14 days of regular water. F991 (15 mg/kg) was administered through i.p. injection daily in conjunction with the DSS treatment cycles, and the same volume of PBS was administered through i.p.in F991-vehicle mice. Mice were sacrificed on day 91 after CAC induction. The level of Igκ and Igλ in the colon tissue or tumor tissues were analyzed by non-reduced western blot. Data were from 4 mice were shown. *ns: no statistical significance* was showed compared with group AOM+DSS+PBS. *AOM+DSS+PBS*: AOM/DSS-induced CAC mice and injected with PBS, *AOM+DSS+F991*: AOM/DSS-induced CAC mice and injected with F991. One representative experiment of two is displayed.
